# Vesico-vaginal Fistule in Europe

**Published:** 1858-11

**Authors:** 


VESICO-VAGINAL FISTULE IN EUROPE.—Dr. Bozeman in a letter to the junior editor, dated Dublin, August 26th, thus writes of the *button-suture* :—

"While in Edinburgh, Dr. Fleming, Surgeon to the Richmond Hospital, found a case with fistule, and announced to the class that I would operate to-day; but much to our disappointment the patient became frightened, and concluded not to submit to the operation. Not a case of this description has ever, as far as I can learn, been cured in Edinburgh. I operated twice while there; once in the Royal Infirmary and once in the practice of Professor Simpson. The case in the Infirmary turned out badly, dying on the sixth day from cellular inflammation. I was in the north of Scotland when this occurred, and you can well imagine my surprise upon returning. Fortunately the parts were preserved for me, and the result of the examination proved satisfactory to all present, viz., that the operation, as far as it was concerned, was entirely successful. The fistule was completely closed, and the suture apparatus was perfect in its adjustment. Not a drop of urine escaped from the time of the operation. The case was a very bad one, but I never performed an operation with which I was better pleased. The cervix uteri was shut up in the bladder, and the operation consisted in restoring it to its natural position in the vagina, and in that way closing the opening. Prof. Simpson and all present were delighted with the procedure. The patient, I have since learned, was one of the greatest drunkards in Edinburgh, and was, several days before the operation, beastly drunk. Under these circumstances, no other termination could well have been expected. I performed an operation in the Royal Infirmary at Glasgow, but have not yet heard from it. I received a letter from Mr. Erichsen, while in Edinburgh, stating that the case which I operated upon in London was cured."

